# The Effect of Longwave Ultraviolet Light Radiation on *Dendrolimus tabulaeformis* Antioxidant and Detoxifying Enzymes

**DOI:** 10.3390/insects11010001

**Published:** 2019-12-18

**Authors:** Wenlong Wang, Chenglong Gao, Lili Ren, Youqing Luo

**Affiliations:** Beijing Key Laboratory for Forest Pest Control, School of Forestry, Beijing Forestry University, Beijing 100083, China; ky2011wwl@163.com (W.W.); gaocl890907@163.com (C.G.)

**Keywords:** ultraviolet light, oxidative stress, antioxidant enzymes, detoxifying enzymes, *Dendrolimus tabulaeformis*

## Abstract

Longwave ultraviolet (UVA) light, in the range of 315–400 nm, has been widely used as a light source in the light trapping of insect pests. Previous studies have demonstrated the oxidative stress and lethal effect of UV radiation on insects. In this study, we evaluated the influence of UVA radiation on the antioxidant and detoxifying enzymes of *Dendrolimus tabulaeformis.* We tested the contents of malondialdehyde (MDA), hydroxyl radical (·OH), hydrogen peroxide (H_2_O_2_), reduced glutathione (GSH), and oxidized glutathione (GSSH) following different exposure time periods of UVA light irradiation on *D. tabulaeformis* adults. In addition, we investigated how the activities of antioxidant and detoxifying enzymes responded to UVA radiation by determining the activities of superoxide dismutase (SOD), catalase (CAT), peroxidase (POD), polyphenol oxidase (PPO), glutathione S-transferase (GST), glutathione reductase (GR), acetylcholinesterase (AChE), carboxylesterase (CarE), alkaline phosphatase (ALP), and acid phosphatase (ACP). Adults were exposed to UVA light for different time periods (0, 5, 15, 30, 60, and 120 min). We found that exposure to UVA light for 5 min resulted in rapid variation in the activities of the antioxidant and detoxification enzyme systems. However, the antioxidant capacity of females was incongruous with that of males following UVA irradiation. Our results confirmed that UVA light irradiation increased the level of oxidative stress and disturbed physiological detoxification in *D. tabulaeformis* adults. Based on the above results, we anticipated that further research of the mechanism of UVA irradiation on the antioxidant and detoxifying enzymes of *D. tabulaeformis* would gain more importance, allowing to develop and use new, less toxic and environmentally friendly pesticides.

## 1. Introduction

*Dendrolimus tabulaeformis* (Lepidoptera: Lasiocampidae) is a serious forest pest in China, which mainly harms *Pinus tabulaeformis* Carr. (Pinaceae), but also feeds on *Pinus densiflora* Siebold and Zucc. (Pinaceae) and *Pinus massoniana* Lamb. (Pinaceae) at the edge of the population distributions [1,2]. *Dendrolimus tabulaeformis* can kill pine forests in blocks following a breakout, seriously endangering the health of the forest and causing huge losses in China. Our previous studies have shown *D. tabulaeformis* displayed conspicuous positive phototactic behavior under light stimulation, and was especially sensitive to UVA light [3].

Longwave ultraviolet (UVA) refers to electromagnetic radiation with a wavelength between 315 and 400 nm. Most moths prefer UVA rays. The use of artificial UVA light has drastically increased in forestry production to control moth pests during the last few decades [4,5,6]. Black light, an artificial type of UVA light, has been widely applied in pest control, especially for nocturnal moths [7,8]. Currently, the light source is transformed mainly from long wavelength light (e.g., sodium vapor lights) to light at a shorter wavelength (e.g., light-emitting diodes (LEDs)) [9]. The detailed responses of the insects are needed to understand the implications of this change for insects.

Under normal conditions, various antioxidants in insect organisms are in a state of dynamic equilibrium to maintain normal physiological activities. UV radiation can generate oxidative stress in insects and destroy the functional activity of protein [10,11]. The toxicity of ultraviolet (UV) light has been reported in various insect pests [12,13,14,15,16,17,18]. To combat against the damage of oxidative stress, insects have evolved an intricate network of enzymatic antioxidant systems [19].

UVA lights, as an environmental stress factor for insects, lead to the production of reactive oxygen species (ROS) [20,21]. Unsaturated fatty acids in biological organisms are highly susceptible to peroxidation, producing toxic lipid peroxides. Antioxidant systems are the bases of a common, basic process occurring in insects. Harman [22] first proposed the theory of free radicals: Polyunsaturated fatty acids produce the free radical intermediate L under the action of free radicals and other oxidative inducers (such as hydroxyl radicals, hydrogen peroxide, or singlet oxygen) and then react with O_2_ to produce LOO and LOOH. They can spontaneously decompose to generate more free radicals to attack other double bonds, produce more lipid peroxide free radicals and, in turn, initiate a chain reaction that generates automatic decomposition. An organism’s antioxidant enzyme enzymatic defense system can react with the free radicals produced by lipid peroxides to terminate the chain reaction. Fridovich [23,24,25] proposed the concept of an antioxidant enzyme system as the result from the incomplete reduction of oxygen representing the primary self-protection mechanisms, including superoxide dismutase (SOD), catalase (CAT), and peroxidase (POD). SOD can catalyze the dismutation of O^2−^ to form H_2_O_2_, and CAT and POD can decompose H_2_O_2_ into H_2_O and O_2_. Glutathione peroxidase (GSH-PX) is coupled to oxidized glutathione (GSSH), which catalyzes the breakdown of oxidized glutathione in glutathione reductase (GR) under the action of the generation of reduced glutathione (GSH).

In the present study, we attempted to evaluate whether UVA light irradiation led to a change in some antioxidant and detoxification enzymes of *D. tabulaeformis* moths. We examined the effects of UVA light irradiation on malondialdehyde (MDA), hydroxyl radical (·OH), hydrogen peroxide (H_2_O_2_), reduced glutathione (GSH), and oxidized glutathione (GSSH) contents. In addition, we studied the antioxidant and detoxifying enzyme activity responses by determining the activities of superoxide dismutase (SOD), catalase (CAT), peroxidase (POD), polyphenol oxidase (PPO), glutathione S-transferase (GST), glutathione reductase (GR), acetylcholinesterase (AChE), carboxylesterase (CarE), alkaline phosphatase (ALP), and acid phosphatase (ACP) in *D. tabulaeformis* adults.

## 2. Materials and Methods

### 2.1. Insects

*Dendrolimus tabulaeformis* pupas were obtained from a pure forest stand of *P. tabulaeformis* in Jianping County, China (41°40′40.79″ N, 119°51′43.19″ E) in mid-July. The pupas were maintained at 25 ± 1 °C and 70% ± 5% relative humidity (RH) with a photoperiod of (16L/8D) and light illumination of 5000 lux in a constant temperature incubator. Adults were used in experiments two days after emergence. Each adult was segregated into 300 mL plastic containers.

### 2.2. UV Irradiation

UVA LED lights (Research and Development Center for Semiconductor Lighting, Chinese Academy of Sciences, Beijing, China), which can emit 365 nm UVA, were used as the source to irradiate *D. tabulaeformis* at 40 μw/cm^2^. The male and female adults were each divided into six individual groups. Each group contained six similarly shaped adults for the experiment. All adults were placed in darkness from 20:00 to 22:00 before irradiation. After the scotophase, six adults per treatment were randomly selected for exposure to UVA for 0 (control), 5, 15, 30, 60, 90, and 120 min. Each treatment was repeated six times. The samples were immediately frozen in liquid nitrogen and stored in a −80 °C freezer for subsequent assays.

### 2.3. Sample Preparation

The wings and thoracic legs were removed, and the remaining moths were then weighed before homogenization of whole moths. The weighed samples were homogenized in ice-cold buffer (0.1 M phosphate buffer, 0.1 mM EDTA-2Na, 10 mM sucrose, 0.9% NaCl, pH = 7.4) at a ratio of 0.1 g of body weight to 1 mL of buffer. The homogenates were centrifuged at 2500× *g* for 20 min at 4 °C, and the supernatant was used for subsequent analysis.

### 2.4. Oxidative Parameters Assay

The oxidative parameters were tested for MDA, ·OH, H_2_O_2_, GSH, and GSSH. These substances, which are intermediate products of oxidation–reduction reactions in respiratory chain systems, were measured using commercially available ELISA kits (Jianglai Biotechnology Co., Ltd., Shanghai, China).

### 2.5. Antioxidant Enzyme Activity Assay

The enzyme activity and related substance contents were determined by the double antibody sandwich method using ELISA kits manufactured by Jianglai Biotechnology Co., Ltd. (Shanghai, China). The measured substances were SOD, CAT, POD, PPO, GST, and GR. Taking SOD as an example, a microplate was coated with a purified SOD antibody to prepare a solid phase antibody, and SOD was sequentially added to the microcapsule of the coated monoclonal antibody. Then, SOD was combined with horseradish peroxidase (HRP)-labeled SOD antibody to form an antibody–antigen–enzyme-labeled antibody complex. The substrate 3,3′,5,5′-tetramethylbenzidine (TMB) was used for color development after thorough washing. TMB was converted to blue under catalysis of the HRP enzyme and converted to its final yellow color via the action of an acid. Color depth was positively correlated with SOD in the sample. The absorbance (OD value) was measured with an enzyme-labeling instrument (SpectraMax Plus 384, Molecular Devices Co., Ltd., Silicon Valley, CA, USA) at a wavelength of 450 nm, and the concentration of SOD activity in the sample was calculated from a standard curve.

### 2.6. Detoxifying Enzyme Activity Assay

The main detoxifying enzymes, including AChE, CarE, ALP, and ACP, were assayed using ELISA kits (Jianglai Biotechnology Co., Ltd., Shanghai, China), according to procedures described in their user manuals.

### 2.7. Data Analysis

All data were analyzed by one-way ANOVA using SPSS 23 (SPSS Inc., Chicago, IL, USA), and multiple comparisons were performed using the LSD method. Statistical results were expressed as mean ± SD, with *p* < 0.05 considered statistically significant, and multiple comparison results were marked by the letter-marking method. Histograms were drawn using GraphPad Prism 7.01 (GraphPad Software Inc., San Diego, CA, USA).

## 3. Results

### 3.1. Oxidative Damage

**Figure 1:**
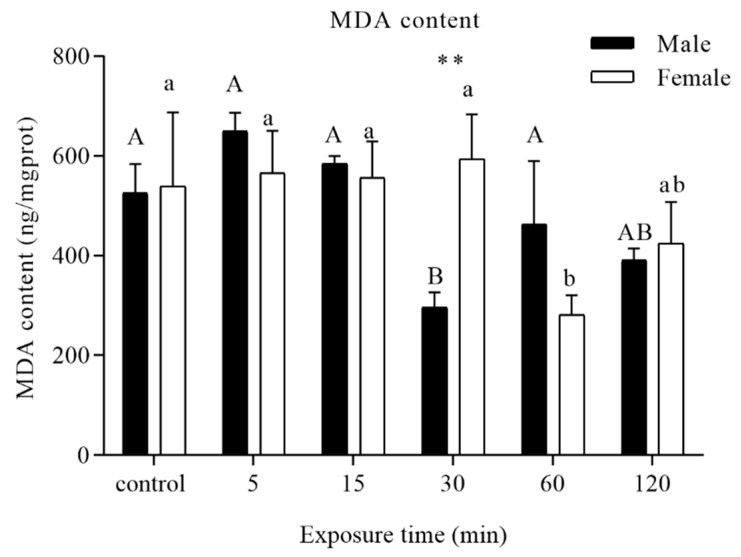
Analysis of different time periods of UVA light irradiation on the malondialdehyde (MDA) content of *D. tabulaeformis* adults. Capital letters designate statistically significant differences between different groups of males, and lowercase letters designate statistically significant differences between different groups of females (*p* < 0.05). Values are the mean ± SD. (*n* = 6). An asterisk designates a statistically significant difference between male and female adults (** *p* < 0.01).

MDA content was chosen as the marker to assess oxidative damage upon adult exposure to UVA light. Compared to the control, a significant decrease was recorded when the males were exposed to UVA lights for 30 min, while a decline appeared at 60 min for females. The MDA content then returned to control levels (Figure 1).

### 3.2. Intermediates of Oxidation–Reduction Reactions

**Figure 2:**
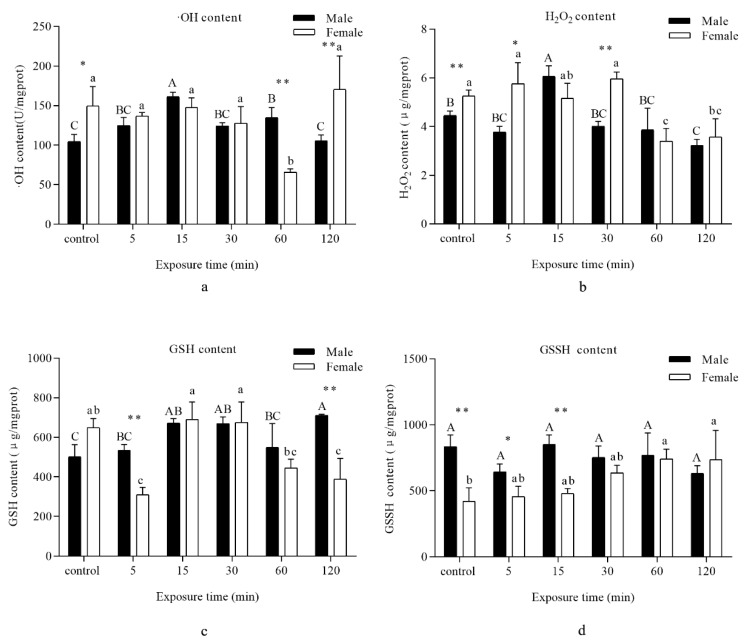
Analysis of different periods UVA light irradiation on the intermediates of oxidation–reduction reactions of *D. tabulaeformis* adults. (**a**–**d)** represent the contents of different intermediates, (**a**)—hydroxyl radical (·OH), (**b**)—hydrogen peroxide (H_2_O_2_), (**c**)—reduced glutathione (GSH), and (**d**)—oxidized glutathione (GSSH) corresponding to the different times. Capital letters designate statistically significant differences between different groups of males; lowercase letters designate statistically significant differences between different groups of females (*p* < 0.05). Values are the mean ± SD. (*n* = 6). An asterisk designates a statistically significant difference between male and female adults (* *p* < 0.05; ** *p* < 0.01).

An evident increase in ·OH and H_2_O_2_ content in males was recorded when the adults were exposed to UVA light for 15 min, and then a significant decrease was found at 120 min. For females, a decrease in ·OH and H_2_O_2_ content was recorded at 60 min (Figure 2a,b).

Compared with the control, GSH quantity was significantly enhanced in males with exposure to UVA light for 15, 30, and 120 min. However, an obvious decrease for females was observed at 5 min, which then reverted to control levels for 15 and 30 min, subsequently dropping again at 60 and 120 min (Figure 2c).

No significant difference in GSSH content was observed when the males were subjected to UVA light, but a distinct ascent was visible for females following exposure to UVA light for 60 and 120 min in comparison with the control (Figure 2d).

### 3.3. Antioxidant Enzymes

**Figure 3:**
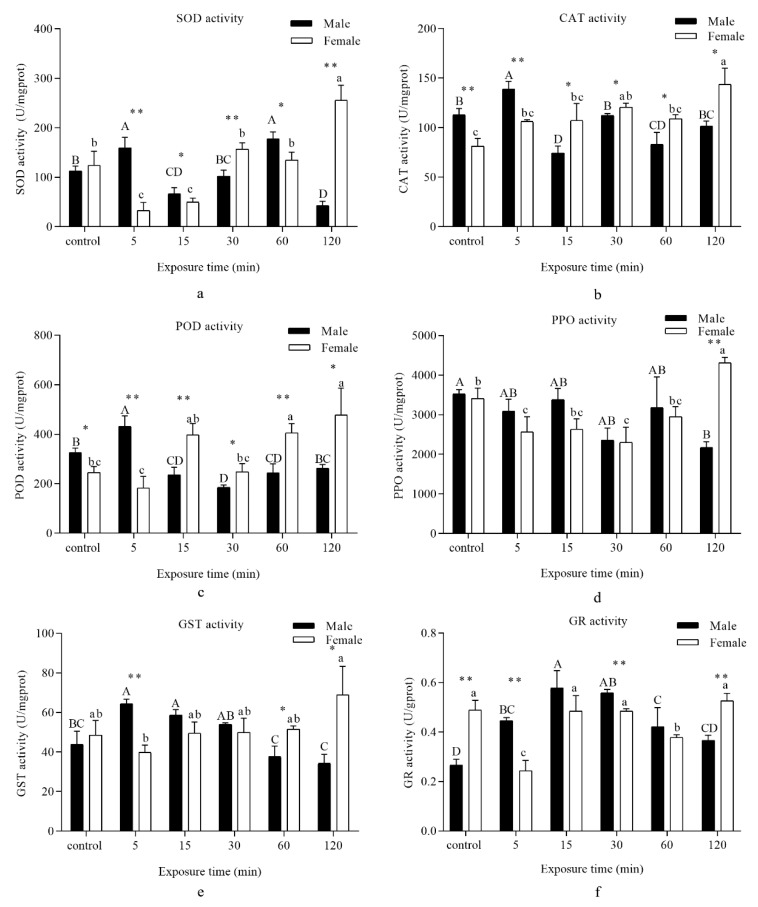
Analysis of different periods UVA light irradiation on the antioxidant enzyme activity of *D. tabulaeformis* adults. (**a**–**f**) represent the activity of different enzymes, (**a**)—superoxide dismutase (SOD), (**b**)—catalase (CAT), (**c**)—peroxidase (POD), (**d**)—polyphenol oxidase (PPO), (**e**)—glutathione S-transferase (GST), and (**f**)—glutathione reductase (GR) corresponding to the different times. Capital letters designate statistically significant differences between different groups of males, while lowercase letters designate statistically significant differences between different groups of females (*p* < 0.05). Values are the mean ± SD. (*n* = 6). An asterisk designates a statistically significant difference between male and female adults (* *p* < 0.05; ** *p* < 0.01).

SOD activity was significantly enhanced in males when they were exposed to UVA light for 5 min compared with the control and drastically declined at 15 min. Afterwards, SOD activity reverted to its baseline at 30 min, while a remarkable increase and a sharp drop were respectively found at 60 and 120 min. However, SOD activity in females displayed different results: A dramatic decrease was found following exposure to UVA light at 5 min in comparison with the control, which then retained an upwards trend until 120 min (Figure 3a).

A remarkable increase in the CAT activity of males was recorded at 5 min, while a significant decrease was recorded at 15 and 60 min compared with the control. As for females, CAT activity presented an upward tendency in comparison with the control, and a marked increase was recorded at 120 min (Figure 3b).

POD activity was significantly increased in males at 5 min and then persistently declined to control levels at 120 min. For females, a marked surge was recorded at 15, 60, and 120 min (Figure 3c).

PPO activity notably declined in males at 30 and 120 min, while a significant decrease was marked at 5 and 30 min. However, after 120 min of exposure, the PPO activity showed a notable increase compared with the control (Figure 3d).

GST activity in males rapidly ascended at 5 and 15 min before decreasing from 60 to 120 min. For females, a striking increase was recorded at 120 min in comparison with the control (Figure 3e).

A gradual upward trend of GR activity was recorded in males when the insects were exposed to UVA light compared with the control. A sharp surge appeared at 15 min and then dropped steadily until 120 min. However, GR activity showed a different tendency in females. A dramatic decline was recorded at 5 and 60 min compared with the control (Figure 3f).

### 3.4. Detoxifying Enzyme

**Figure 4:**
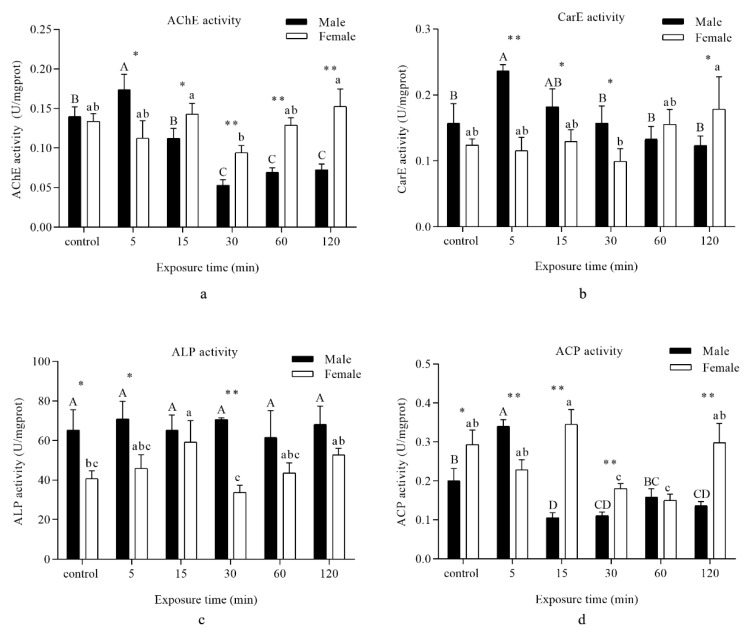
Analysis of different periods of UVA light irradiation on the detoxifying enzyme activity of *D. tabulaeformis* adults. (**a**–**d**) represent the activity of different enzymes, (**a**)—acetylcholinesterase (AChE), (**b**)—carboxylesterase (CarE), (**c**)—alkaline phosphatase (ALP), and (**d**)—acid phosphatase (ACP) corresponding to the different times. Capital letters designate statistically significant differences between different groups of males; lowercase letters designate statistically significant differences between different groups of females (*p* < 0.05). Values are the mean ± SD. (*n* = 6). An asterisk designates a statistically significant difference between male and female adults (* *p* < 0.05; ** *p* < 0.01).

A significant increase in AChE activity in males was marked when insects were exposed to UVA light for 5 min, and then a remarkable decrease was observed for 30–120 min of UVA light irradiation. However, no significant difference in AChE activity was recorded when the females were subjected to UVA light compared with the control, and a significant decrease was found after UVA light exposure for 120 and 90 min (Figure 4a).

CES activity was remarkably increased in males at 5 min. As for females, no significant difference in CES activity was noted when insects were subjected to UVA light in comparison with the control, and a significant decrease was found after UVA light exposure for 120 and 90 min (Figure 4b).

No remarkable difference in ALP activity was observed when the males were subjected to UVA light. However, a distinct ascent was observed in females following exposure to UVA light for 15 min in comparison with the control (Figure 4c).

ACP activity was significantly enhanced in males when they were exposed to UVA light for 5 min compared with the control, and a notable decline was observed at 15 min. Afterwards, ACP activity reverted to baseline levels following 60 min of irradiation. However, ACP activity in females showed a different trend. A drastic decrease was found following exposure to UVA light for 30 and 60 min in comparison with the control, and there was an upward tendency up to 120 min of exposure (Figure 4d).

## 4. Discussion

MDA has been used as a biological marker of oxidative stress [26,27]. In our study, UVA light radiation did not generate a significant increase but produced a drop in the level of ROS damage to lipids in *D. tabulaeformis* adults. The fluctuation of MDA might be due to the activities of antioxidant enzymes, which eliminated excess lipid peroxidation, while MDA returned to a normal level.

Intermediates of the oxidation–reduction system, with the exception of GSSH content, presented a trend of increase and then fall back. The consequence of this was that the insect system combated against the effect of UVA irradiation pressure to rehabilitate redox balance. The effects of UVA light may be weaker than those of UVB light radiation [28,29]. Under normal conditions, glutathione exists in GSH and GSSH forms. When free radicals increase, a large amount of GSSH accumulates in the cells, which disrupted the homeostasis. GSH scavenges free radicals through the transfer of electrons and protons, while GSSH could not eliminate free radicals and only participated in maintaining two forms of glutathion Glycine’s homeostasis [30,31].

SOD is an important antioxidant protein used to mitigate excessive levels of intracellular superoxide radicals. In the present study, the changes in SOD activity showed mixed results, suggesting that UV light irradiation induced superoxide radical formation in *D. tabulaeformis* adults, as had been observed in the Antarctic midge *Belgica antarctica* [32]. SOD activity significantly increased when the insects were exposed to UV light for 5 min, suggesting that SOD was stimulated by scavenging superoxide radicals to protect adults from UVA stress. An increase in SOD activity, which was likely a response to increased ROS formation, had also been reported [33]. However, the females showed an opposite trend from 5 until 30 min, and then maintained an upward tendency until 120 min. According to the theory of life history, reproductive sustainability was a crucial determinant of life [34,35]. We presumed that there was another regulation control system that responded to oxidative stress. Previous studies also showed that UVA increased the fecundity and oviposition rate of *Mythimna separata* [36]. With the increase in UVA irradiation period, SOD activity reverted to its baseline levels for 30 min, while a remarkable increase and a sharp drop were respectively found for 60 and 120 min. It was not consistent with previous reports showing that high doses of UV irradiation suppressed the activity of protective enzymes cells [37,38].

CAT is known to reduce high amounts of H_2_O_2_ [25]. In examining the H_2_O_2_ contents (Figure 2b), when the amount of H_2_O_2_ significantly increased at 15 min, the activity of CAT was obviously lower than in other groups. During the whole time periods, the results showed a negative correlation between the amount of H_2_O_2_ and activity of CAT. We assumed that an increase in CAT activity would result in a decrease in H_2_O_2_ concentration. Previous studies have shown that CAT protected insects against oxidative stress [39].

When *D. tabulaeformis* adults were exposed to UVA light for 5 min, a significant increase in POD activity functioned to maintain the balance of H_2_O_2_ components. However, exposure to UVA light for longer times resulted in a decrease in enzyme activity. POD may be associated with the scavenging of H_2_O_2_ [40]. The control strategies were similar to CAT activities as explained above. By contrast, PPO showed another outcome of irradiation. Previous studies have shown that enzyme activity can be decreased by negative feedback from excess substrate or damage by oxidative modification [41]. A significant increase in CAT activity in response to UV light irradiation at a longer exposure time and a simultaneous decrease in POD activity suggested that CAT may have a more important role than POD in the scavenging of H_2_O_2_ under longer exposure times.

GST and GR, considered to be primary antioxidant enzymes, are effective in metabolizing lipid peroxides [42]. In our study, we considered whether increased levels of GST and GR would lead to removal of the lipid peroxidation products that accumulated due to UVA light exposure of *D. tabulaeformis* adults. GSH had some other metabolic pathways. For example, Ganguli found another glutathione metabolizing enzyme protein complex that can degrade GSH in *Saccharomyces cerevisiae* [43], the thioredoxin system can also replace GR to produce GSH [44,45]. We believed that the decrease in GSH in the results was the result of the combined action of multiple metabolic pathways, not just the catalytic effect of GR. In addition, GR activity was at a relatively low level compared to other enzymes, which may also cause other alternative pathways in the body to be activated, so GSSH remained at a high level. Our results regarding MDA concentrations also confirmed that this is indeed the case.

AChE, a widely occurring detoxifying enzyme, plays an important role in regulating the normal conduction of nerve impulses between synapses and maintaining the normal physiological functions of the organism [46]. AChE is generally considered to be the target of organophosphorus and carbamate insecticides, and its amino acid changes are more likely to cause insect resistance to insecticides [47]. In addition, AChE also plays an important role in the growth and development of insects. Previous studies have shown that with the gene silencing of AChE, the fecundity of *Helicoverpa armigera* was drastically reduced [48]. In our study, the AChE activities in males and females were both suppressed after UVA irradiation for 30 min. The reason for this phenomenon needs further research. CES and ALP activity in *D. tabulaeformis* showed the same tendency as AChE. We thus suspect that UVA irradiation may prevent neurotransmitter transmission in insects. UVA light irradiation hardly influences ALP activity. Both ALP and ACP belong to the nonspecific phosphohydrolase involved in the transfer and metabolism of phosphate groups. The optimum pH is >7 for ALP and <7 for ACP [49]. These values also implied that UVA light irradiation increased the production of ROS and disturbed the acid–base balance in *D. tabulaeformis.*

## 5. Conclusions

In conclusion, UVA light irradiation disturbed the inward redox balance in *D. tabulaeformis* moths following continuous exposure. The UVA irradiation caused oxidative stress in *D. tabulaeformis* moths. With the accumulation and overproduction of ·OH and H_2_O_2_, the protective functions of antioxidant and detoxifying enzymes in the *D. tabulaeformis* moths were activated to defend these stress. The SOD, CAT, POD, AChE, and ACP activities presented relatively large fluctuations during the irradiation period. On the other hand, the ALP activity was basically stable at an equilibrium level, demonstrating that the UVA irradiation caused the accumulation of ROS. The enzymes activities between males and females presented consistency during the experiment. We speculated that as females were responsible for procreation, they may endure more stress from the external environment.

This study focused solely on antioxidant and detoxifying enzymes during UVA light irradiation. Further research should clarify the specific processes of related substances in synthesis and metabolism, and then develop new low-toxic, environmentally friendly pesticides.
